# Supplementary material for: HIVprotI: an integrated web based platform for prediction and design of HIV proteins inhibitors
Source: J Cheminform. 2018 Mar 9;10:12. doi: 10.1186/s13321-018-0266-y (PMC5845081; doi:10.1186/s13321-018-0266-y)
Supplement: Supplementary file 2 — Additional file 2. Source code of HIVProtI web server. [file 13321_2018_266_MOESM2_ESM.zip › HIVprotI_Source-code/hivprotI/weka-3-6-6/doc/weka/gui/beans/WekaWrapper.html]

WekaWrapper


---


|  |  |  |
| --- | --- | --- |
| **Overview** \| **Package** \| **Class** \| **Tree** \| **Deprecated** \| **Index** \| **Help** \| **Weka's home** | |  |
| **PREV CLASS**   NEXT CLASS | **FRAMES**    **NO FRAMES**     **All Classes** |  |
| SUMMARY: NESTED \| FIELD \| CONSTR \| METHOD | DETAIL: FIELD \| CONSTR \| METHOD |  |


---


## weka.gui.beans Interface WekaWrapper

**All Known Implementing Classes:**: Associator, Classifier, Clusterer, Filter, Loader, Saver

---

``` public interface WekaWrapper ```

Interface to something that can wrap around a class of Weka
algorithms (classifiers, filters etc). Typically implemented
by a bean for handling classes of Weka algorithms.

**Since:**
:   1.0

**Version:**
:   $Revision: 1.4 $

**Author:**
:   Mark Hall

---

| **Method Summary** | |
| --- | --- |
| `java.lang.Object` | `getWrappedAlgorithm()`             Get the algorithm |
| `void` | `setWrappedAlgorithm(java.lang.Object algorithm)`             Set the algorithm. |

**Method Detail**

### setWrappedAlgorithm

```
void setWrappedAlgorithm(java.lang.Object algorithm)
```

:   Set the algorithm.

    :   **Parameters:**: `algorithm` - an `Object` value **Throws:**: `java.lang.IllegalArgumentException` - if the supplied object is not of the class of algorithms handled by this wrapper.

---


### getWrappedAlgorithm

```
java.lang.Object getWrappedAlgorithm()
```

:   Get the algorithm

    :   **Returns:**: an `Object` value


---


|  |  |  |
| --- | --- | --- |
| **Overview** \| **Package** \| **Class** \| **Tree** \| **Deprecated** \| **Index** \| **Help** \| **Weka's home** | |  |
| **PREV CLASS**   NEXT CLASS | **FRAMES**    **NO FRAMES**     **All Classes** |  |
| SUMMARY: NESTED \| FIELD \| CONSTR \| METHOD | DETAIL: FIELD \| CONSTR \| METHOD |  |


---
